# Exploring Hedonic and Eudaimonic Items of Well-Being in Mediterranean and Non-Mediterranean Countries: Influence of Sociodemographic and Lifestyle Factors

**DOI:** 10.3390/ijerph19031715

**Published:** 2022-02-02

**Authors:** Vanda Andrade, Stefano Quarta, Marta Tagarro, Lence Miloseva, Marika Massaro, Mihail Chervenkov, Teodora Ivanova, Rui Jorge, Viktorija Maksimova, Katarina Smilkov, Darinka Gjorgieva Ackova, Tatjana Ruskovska, Elena Philippou, Georgia Eirini Deligiannidou, Christos A. Kontogiorgis, María-Teresa García Conesa, Paula Pinto

**Affiliations:** 1 Instituto Politécnico de Santarém, Escola Superior Agraria, 2001-904 Santarem, Portugal; vanda.andrade@esa.ipsantarem.pt (V.A.); rui.jorge@esa.ipsantarem.pt (R.J.); 2 Laboratory of Biochemistry and Molecular Biology, Department of Biological and Environmental Sciences and Technologies, University of Salento, 73100 Lecce, Italy; stefano.quarta3@unisalento.it; 3 Instituto Politécnico de Santarém, Escola Superior de Educação, 2001-902 Santarem, Portugal; marta.tagarro@ese.ipsantarem.pt; 4 Faculty of Medical Sciences, University Goce Delcev, Str. Krste Misirkov, No. 10-A, POB 201, 2000 Stip, North Macedonia; lence.miloseva@ugd.edu.mk (L.M.); viktorija.maksimova@ugd.edu.mk (V.M.); katarina.smilkov@ugd.edu.mk (K.S.); darinka.gorgieva@ugd.edu.mk (D.G.A.); tatjana.ruskovska@ugd.edu.mk (T.R.); 5 National Research Council (CNR), Institute of Clinical Physiology, 73100 Lecce, Italy; marika@ifc.cnr.it; 6 Faculty of Veterinary Medicine, University of Forestry, 1797 Sofia, Bulgaria; vdmchervenkov@abv.bg; 7 Slow Food in Bulgaria, 9 Pierre De Geytre St. bl. 3, 1113 Sofia, Bulgaria; tai@bio.bas.bg; 8 Department of Plant and Fungal Diversity and Resources, Institute of Biodiversity and Ecosystem Research, Bulgarian Academy of Sciences, 1113 Sofia, Bulgaria; 9 Life Quality Research Centre (CIEQV), IPSantarém/IPLeiria, 2040-413 Rio Maior, Portugal; 10 Centro de Investigação Interdisciplinar Egas Moniz (CiiEM), Instituto Universitário Egas Moniz, 2829-511 Monte de Caparica, Portugal; 11 Department of Life and Health Sciences, School of Sciences and Engineering, University of Nicosia, Nicosia 1700, Cyprus; philippou.e@unic.ac.cy; 12 Department of Nutritional Sciences, King’s College London, London SE1 9NH, UK; 13 Laboratory of Hygiene and Environmental Protection, School of Medicine, Democritus University of Thrace, Dragana, 68100 Alexandroupolis, Greece; edeligia@med.duth.gr (G.E.D.); ckontogi@med.duth.gr (C.A.K.); 14 Research Group on Quality, Safety and Bioactivity of Plant Foods, Centro de Edafología y Biología Aplicada del Segura-Consejo Superior de Investigaciones Científicas (CEBAS-CSIC), Campus de Espinardo, 30100 Murcia, Spain

**Keywords:** life satisfaction, subjective well-being, hedonic well-being, eudaimonic well-being, lifestyle habits, Mediterranean

## Abstract

Increased understanding of subjective well-being (SWB), as well as factors that influence it, are essential to enhance well-being at the individual and national level. We have applied a hedonic and eudaimonic 9-item composed tool (SWB score) to measure SWB across several Mediterranean (MED) and non-Mediterranean (non-MED) countries, and to explore the association between the SWB score and a range of sociodemographic, health and Mediterranean lifestyle factors. A specifically designed web-based questionnaire was distributed to adult participants (N = 2400) from Spain, Italy, Portugal, Bulgaria and Republic of North Macedonia. Results showed that the SWB score was significantly different across the examined countries with the MED participants displaying slightly higher average scores than the non-MED ones (6.3 ± 1.5 vs. 6.1 ± 1.6, *p* = 0.002). Several sociodemographic, health status and lifestyle factors displayed a significant but limited association with the 9-item SWB score, with a multiple regression model explaining around 17% of the variance. Nevertheless, our results support that a closer adherence to Mediterranean lifestyle habits—the Mediterranean Diet, spending time with friends, family, and in nature, being active, and getting adequate rest at night—has a positive influence on the 9-item SWB score. Further research is needed to advance the understanding of the measuring and differentiating of SWB across different populations and to establish all the factors that influence it.

## 1. Introduction

Enhancing the well-being of the population in the European Region became one of the key targets in 2015 [1]. Well-being has been defined by Ryan and Deci in 2001 as “optimal psychological functioning and experience” [2], and later by the Organization for Economic Co-operation and Development (OECD) as “good mental state, including all of the various evaluations, positive and negative, that people make of their lives, and the affective experiences of people to their experiences” [3]. The World Health Organization (WHO) has included the concept of well-being in the definition of positive mental health: “a state of well-being in which the individual realizes his or her own abilities, can cope with the normal stresses of life, can work productively and fruitfully, and is able to make a contribution to his or her community” [4]. In a broad sense, Diener has highlighted well-being as a multidimensional concept, covering how well individuals are doing in life, including social, health, economical, and subjective dimensions [5]. Some objective indicators used to assess well-being at a population level are education, job availability, income, housing, and safety. However, well-being is influenced by cultural factors and values, traditions, and beliefs, and therefore must also be evaluated through qualitative indicators and subjective experiences of well-being [6].

In the study of subjective well-being, two main domains have emerged: the hedonic and the eudaimonic domains [2,7]. The hedonic well-being concerns the experience of pleasure vs. displeasure and includes judgments about the good/bad elements of life, often summarized as happiness [2,5,8]. Most research within the hedonic view adopted the term “subjective well-being” (SWB), with assessment of three components: (i) life satisfaction, (ii) the presence of positive mood, and (iii) the absence of negative mood [2,5,9]. The eudaimonic well-being refers to living in accordance with the “daimon”, an ideal in the sense of being an excellence, a perfection toward which one strives, thus giving meaning and direction to one’s life [10]. The term “psychological well-being” (PWB) has been used in research within this approach, with assessment of positive relationships with others, one’s sense of mastery and personal growth, self-acceptance, autonomy, and a sense of purpose in life [2,11,12]. Most researchers agree that well-being is a multidimensional concept, and that to fully understand individuals’ subjective perceptions of life, measuring instruments should include in a coherent measure hedonic general evaluation of life satisfaction, hedonic negative and positive affect states, and eudaimonic dimensions, such as purpose and meaning of life, and psychological functioning [2,3,7,11,13]. Results from the European Social Survey (ESS round 6) showed that hedonic and eudaimonic well-being are distinct concepts, but strongly correlated [14], supporting the improvement of instruments with hedonic and eudaimonic indicators.

Regardless of the philosophy, hedonic and eudaimonic well-being have been long associated with many benefits including a better general health status, decreased risk of non-communicable diseases (NCDs), and increased life expectancy [5,7,8,15,16,17]. Lifestyle habits considerably influence our health; in particular, the Mediterranean lifestyle is considered a way of life that can effectively promote not only physical health but also social and mental well-being [18]. Between the components of the Mediterranean lifestyle, a lot of research provided evidence of the favorable influence of the Mediterranean diet (MD) on the prevention of NCDs including neurodegenerative and neuropsychological disorders [19,20]. More recently, a positive impact of the adherence to MD and/or consumption of some of their main healthy food (i.e., fruits and vegetables) on subjectively experienced well-being has also been repeatedly reported [21,22,23,24,25,26]. However, the data are still limited, and the results are not yet conclusive, as many of those studies have been conducted in specific subpopulations from individual countries (i.e., Spain, Portugal) or with specific characteristics (e.g., body mass index, BMI; age, etc.). Further, the investigators applied different measuring instruments/items to assess MD adherence and subjective or psychological well-being, and thus, comparison between studies and/or between different populations is not trivial.

On the other hand, the Mediterranean lifestyle comprises not only specific dietary components and food habits, but also other potentially protective factors including moderation and conviviality of food consumption (e.g., sharing meals), physical activity (daily activities at home or at the workplace, leisure and outdoor activities), adequate rest (nocturnal sleep, short day naps “siesta”), social relationships with friends, family, etc. [18,27,28]. To study MD adherence and other lifestyle components across different European Mediterranean (MED) and non-Mediterranean (non-MED) countries, as well as its association with some hedonic and eudaimonic indicators of well-being, we designed a questionnaire (MeDiWeB, Mediterranean Diet and Well-Being [29,30]) containing questions about: (i) sociodemographic factors; (ii) Mediterranean lifestyle and MD adherence, using a cross-national validated version of the 14-MEDAS (Mediterranean Diet Adherence Screener) [31], and (iii) hedonic and eudaimonic indicators of well-being. In a first step, we used the dietary habits section of the MeDiWeB survey to assess and compare MD adherence across different European MED and non-MED countries. Our results corroborated that the MED participant countries had a slightly better adherence to the MD than the non-MED ones but that, in general, the values indicated a persistent moderate to weak adherence in participants from all the countries included in the study, as well as rather small differences across countries [30]. In the present paper, we expand our research by exploring the relationship between a range of sociodemographic, health related and Mediterranean lifestyle factors, including MD adherence, and some features of the hedonic and eudaimonic perceptions of life. Under the OECD subjective well-being framework [3], questions on hedonic life evaluation, hedonic affect (both positive and negative), and eudaimonia were included in the MeDiWeB survey. We adopted the five core questions recommended by OECD [3]: evaluation of global life satisfaction, feeling happy, feeling worried and feeling depressive (hedonic indicators), and evaluation of worthwhile life (eudaimonic indicator). In line with recent research evidence linking stress and energy with well-being [32,33], six more indicators were used: feeling nervous/stressed, feeling tired (hedonic indicators), unable to cope, confident to handle problems, feeling energetic, and efficient (eudaimonic indicators) [3,34,35]. Thus, the specific objectives of the present study were:

(1) to evaluate SWB in participants from various European MED and non-MED countries, using the same common measuring instrument that combines a range of hedonic and eudemonic indicators all included in the MeDiWeB questionnaire;
(2) to explore the relationship between SWB and a series of sociodemographic and health related factors as well as Mediterranean lifestyle habits, including MD adherence.

## 2. Materials and Methods

### 2.1. Study Design and Ethics

The current study is part of a research project carried out under the frame of MeDiWeB (Mediterranean Diet and Well-Being) consortium [29,30,31] which was designed to assess MD adherence and other Mediterranean lifestyle characteristics across participants of three MED (Spain, SP; Italy IT; Portugal PT) and two non-MED countries (Bulgaria, BG; Republic of North Macedonia, NMK), and to explore their association with SWB. For this purpose, we developed and applied an online questionnaire (MeDiWeB questionnaire) with 57 questions divided into three sections [29]. The first section consisted of an introductory explanation of the study and the nature of the participation, with a question at the end asking for authorization to use the data anonymously for statistical analysis and scientific publication. After consenting to fill the questionnaire, the participants proceeded to the second section which included a number of questions about sociodemographic characteristics, lifestyle habits, health related characteristics (BMI and diagnosed pathology), and SWB. The third section was devoted to dietary habits and included, among others, the 14 items used to calculate MD adherence (14-MEDAS score) [31]. The questions were structured following the OECD recommendations regarding subjective self-reporting, specifically, inclusion of subjective evaluation questions at or near the beginning of the survey, to avoid any potential bias effects [3]. The questionnaire was approved by the Ethics Committee of each partner research institution and complies with European Regulation on Data Protection [36]. A full English version of questionnaire can be found at [29] within the Supplementary Materials section.

With regards to SWB, a total of 11 questions were assessed using a 11-point Likert-type scale, where 0 = not at all to 10 = completely/all the time. The questions were classified as hedonic or eudaimonic according to OECD [3] as follows: (1) Life satisfaction (hedonic, cognitive evaluation)—overall, how satisfied are you with your life as a whole these days?; (2) Worthwhile life (eudaimonic)—to what extent do you feel that the things you do in life are worthwhile; (3) Feeling happy (hedonic, positive affect)– how happy did you feel during the last week?; (4) Feeling worried (hedonic, negative affect)- how worried did you feel during the last week?; (5) Feeling depressed (hedonic, negative affect)- did you feel depressed during the last week?; (6) Feeling nervous and stressed (hedonic, negative affect)- during last week, how often did you feel nervous and stressed?; (7) Unable to cope (eudaimonic)—during last week, how often did you feel that you were unable to cope with all the things you had to do?; (8) Confident to handle problems (eudaimonic)- during last week, how often did you feel confident about your ability to handle your personal problems?; (9) Energetic (eudaimonic)- last week, how energetic did you normally feel in the middle of the day?; (10) Tired (hedonic, negative affect)—last week, how tired did you normally feel in the middle of the day?; (11) Efficient (eudaimonic)– last week, how efficient did you normally feel in the middle of the day?

### 2.2. Data Collection

The questionnaire was prepared in the national language of each of the participating countries, and was disseminated through institutional mailing lists, social media, personal contacts, and word-of-mouth communication for the collection of data. The questionnaire was confidential and filled anonymously online. Data were collected between April 2019 and mid-March 2020 (before the COVID-19 lockdown). Participants who indicated (i) lack of consent, (ii) age < 18 years, (iii) duplicates, and/or (iv) participants whose nationality differed from the country in which they were living in, were eliminated from the study. A total of 2400 adults (age ≥ 18 years) distributed across SP (N = 485), PT (N = 484), IT (N = 505), NMK (N = 434) and BG (N = 492) were finally included in the analysis.

### 2.3. Statistical Analysis

The variables were qualified as nominal, ordinal and continuous variables and tested for normality and heteroscedasticity (Kolmorogov–Smirnoff and Levene tests) rendering the use of non-parametric analysis as the best choice [37]. We present and discuss the results of scale variables using the mean ± standard deviation (SD), as well as the median and interquartile range (IQR). Absolute frequencies and percentages are used to represent ordinal or nominal variables. We additionally estimated the standardized mean difference (SMD) as (mean score MED group—mean score non-MED group)/total sample SD [38]. Mann–Whitney and Kruskal–Wallis tests were used to assess differences between MED and non-MED groups of countries, and between countries, respectively, for continuous variables; Chi-squared tests were used for nominal and ordinal variables. All statistical tests were based on two-sided tests (bilateral significance) with a significant level of 5% (α = 0.05).

Exploratory factorial analysis of the SWB items with Varimax rotation and Kaiser Normalization was used for data reduction, and components with eigenvalues above 1 were retained. The number of components to retain was also based on scree plot analysis, the variance explained by the factor model, and the pattern of factor loadings [39]. The adequacy of data for the factor analysis was evaluated by Keiser–Meyer–Olkin Measure of Sampling Adequacy (KMO < 0.5 unacceptable; 0.51 to 0.60 poor, but acceptable; 0.61 to 0.70 mediocre; 0.71 to 0.80 moderate; 0.81 to 0.90 good; 0.91 to 1.00 excellent), and Bartlett’s Test of Sphericity (*p* < 0.001) [40]. Reliability was assessed with Cronbach’s alpha (<0.5 unacceptable; 0.51 to 0.60 poor; 0.61 to 0.70 questionable; 0.71 to 0.80 acceptable; 0.81 to 0.90 good; 0.91 to 1.00 excellent), and Composite Reliability (CR > 0.7) [41]. Convergent Validity was assessed by Average Value Extracted (AVE, >0.5 acceptable; >0.7 very good) [41]. All questions were coded in the same direction, thus, an inverted scale, 10–“x”, was applied to the following questions: feeling worried; feeling depressed; feeling nervous and stressed; unable to cope. A final 9-item tool was considered reliable to use across the participant countries to estimate a SWB score.

To investigate the potential association between the SWB score (dependent variable) and the different sociodemographic, health status and lifestyle factors included in this study (independent variables), we first estimated non-parametric partial Spearman correlation coefficients (*ρ*) adjusted in each case for the corresponding confounding variables. The confounders were identified by significant (*p* < 0.05) bivariate correlations between the SWB score and each of the studied factors: sex, age, nationality, education level, marital status, number of meals per day, smoking, sleeping hours at night, time spent in nature, time spent with family, time spent with friends, sharing meals, daily normal activity, leisure activity, sport practicing, BMI, pathology and 14-MEDAS score. Data are presented as the Spearman’s partial correlation values with their corresponding *p*-values. Since conducting multiple analyses on the same dependent variable may result in increased chance of committing a Type I error, the *p*-value adjusted by Bonferroni’s Correction is additionally indicated (*p*-value < 0.0025) [42].

We next wanted to further test the hypothesis of how the different independent variables (factors) may act together to affect the dependent variable (SWB score), and to confirm the nature (positive or negative) and significance of the relationship. For this purpose, we used those variables first identified as statistically correlated with the 9-item score (*p* < 0.05) to enter them into a multiple linear regression model, conducted using the backward stepwise method.

We carried out all statistical analyses using the Statistical Package for the Social Sciences (SPSS) version 26.0 statistical package for Windows (SPSS, Inc., Chicago, IL, USA).

## 3. Results

### 3.1. Sample Sociodemographic and Lifestyle Characteristics

The comparative analyses of the sociodemographic and lifestyle characteristics of MED and non-MED participants are included in Table 1 and Table 2, respectively, (data of each of the individual countries are presented in Supplementary Tables S1 and S2). Overall, most of the evaluated characteristics were significantly different between the MED and the non-MED groups (most *p*-values < 0.001). The global sample population (N = 2400) was constituted by a higher proportion of women (66.6%) than men (33.3%) (Table 1). Participants were, on average, ≈38.0 years old with the oldest participants from MED countries (40.1 ± 14.2), and the youngest ones from non-MED countries (36.7 ± 13.7). Around 52% of the population was married or in an analogous relationship, and the number average household members was between three and four. Most of the participants had a high education level, with higher percentage for MED participants (79.5%) than non-MED ones (61.9%). The majority of the participants were employed (≈65%), mostly having “white collar” jobs (professional, desk, managerial, administrative work, etc.). Most of the participants were healthy (66.7 to 86% reported not to have a diagnosed pathology) and were in the normal weight BMI class (≈24 kg/m^2^; normal weight between 18.5 and 24.9 kg/m^2^).

Regarding lifestyle characteristics, most participants were non-smokers (≈65–85%) and had sedentary habits (Table 2). More than 80% of the participants reported to have a low daily activity. At leisure time, most of the participants preferred relaxing activities or activities that did not require physical efforts. Only 27.3% of the participants from MED countries and 11.6% from non-MED countries practiced a more intense activity. Most participants (67% and 79%) declared to spend between 6 and 8 h of nocturnal sleep while 31% to 64% slept some “siesta” (short daytime naps) with MED participants from Spain exhibiting the highest frequency of these naps (Supplementary Table S2). Most of the sample population distributed their spare time between family (frequently), friends and nature (sometimes) with small differences between MED and non-MED participants.

With regard to eating habits, most of the participants declared sharing their meals with family or friends, with this proportion being higher in the MED participants (83.8%) than in the non-MED ones (75.8%). There were also significant differences between these two groups in the frequency of meals per day, with three to five meals per day in the MED representatives and two to four meals per day in the non-MED ones. The data of the 14-MEDAS score have been reported in a previous study [30] and are included here as variables for further comparative and correlation analyses. Overall, MED and non-MED participants showed moderate (7.34 ± 1.85) and weak (5.57 ± 1.82) MD adherence, respectively (*p*-value < 0.001).

### 3.2. General Overview of SWB

Table 3 collects the scoring for the 11 hedonic and eudaimonic items evaluated in this study in the overall sample and in the MED and non-MED groups of participants (results for individual countries are presented in Supplementary Table S3). Positive perceptions of eudaimonic and of hedonic items presented median scores between 6 and 8 points of the 0 to 10 Likert-type scale (Table 3). The highest scores were observed for the eudaimonic meaning item “worthwhile life” and the hedonic evaluative item “life satisfaction”, with the highest scores observed in the MED participants (worthwhile life, 7.84 ± 1.46, followed by life satisfaction, 7.48 ± 1.50). Negative perceptions of eudaimonic functioning and hedonic affect presented a higher variation, with median scores between 2 and 6 (Table 3). The lowest scores were observed for the responses to “feeling depressed” (3.17 ± 2.76 in the MED group of countries, and 3.36 ± 3.07 in the non-MED group).

Comparison between the MED participants vs. the non-MED participants shows that the differences between these two groups of participants reached statistical significance only for some items (eudaimonic: worthwhile life, efficient, energetic; hedonic: life satisfaction, happy, worried). Higher values were observed in MED participants for all these items, but the magnitude of these differences was small. We estimated the standardized mean difference (“effect size”) between the MED and non-MED groups that ranged from a minimum difference of −0.02 for the item “feeling tired” up to a maximum difference of 0.5 for the item “life satisfaction” (Table 3).

### 3.3. SWB 9-Item Tool: Properties and Estimation of the SWB Score

We next investigated the combination of those 11 items into a pooled SWB tool that could be used to estimate a SWB score and to analyze the relationships between the SWB score and different factors. An exploratory factorial analysis with the 11 items was first performed, resulting in three extracted components. The items ‘confident to handle problems’ and ‘tired’ did not load uniquely in any of the extracted components, both for the global sample and for each of the individual countries. In addition, these two items displayed a different distribution in the three extracted domains among the countries and thus, both items were removed from the set of items used to construct the pooled item score [43]. The analysis proceeded with the remaining 9 items, which showed good adequacy to the factor model (Table 4). Factor extraction rendered two components that summarized 61.8% of total variance (Table 4). Component 1 (C1) accounted for 31.7% of total variance, with high loadings of the items related to positive perceptions: life satisfaction, worthwhile life, feeling happy, energetic, and efficient. Component 2 (C2) accounted for 30.1% of total variance and showed high loadings of the items related to negative perceptions: feeling worried, feeling depressed, feeling nervous and stressed, and being unable to cope (Table 4). Internal consistency, composite reliability, and convergent validity of the 9-item pooled score and components were considered adequate (Table 4). Factor analysis was also performed with individual countries to ascertain that the 9-item pooled score could be used to compare the countries participating in the present study. Analysis rendered the same two components, explaining more than 50% of total variance, and the same distribution of items between the two extracted components was observed among all countries (Supplementary Table S4).

We used the SWB 9-item tool to estimate the SWB score for each country (Supplementary Table S5) and to compare MED and non-MED participants (Table 5). Globally, the mean SWB 9-item score was 6.2 ± 1.5, with participants from MED countries displaying slightly but significantly higher scores than the non-MED countries (6.3 ± 1.5 vs. 6.1 ± 1.6, *p* = 0.002). We additionally estimated the partial scores for the C1 and C2 components. Participants from the MED countries also showed a higher score for the pooled positive perceptions (C1) than participants from the non-MED countries (7.0 ± 1.3 vs. 6.5 ± 1.6, *p* < 0.0001). No significant differences were found for the pooled negative perceptions (C2) between participants from MED and non-MED countries (*p* = 0.115).

### 3.4. Correlations Analyses and Multiple Regression Model: Association between the SWB Score and the Sociodemographic, Health Status and Lifestyle Factors

The results of the non-parametric partial correlations between the sociodemographic characteristics, health status related factors, and Mediterranean lifestyle factors, and the 9-item SWB score are included in Table 6. Of the 20 independent variables included in the analyses, six of them were not significantly correlated with the pooled SWB score (education level, household, siesta, meals per day, sharing meals and BMI; *p*-value > 0.05). From the fourteen significantly correlated with the pooled SWB score, six attained a *p*-value < 0.0025 (below the Bonferroni cut-off level) emphasizing that higher values of the SWB score were positively associated with older ages, having a full-time job, being a man, not having a pathology, longer sleeping time at night, and more time spent with friends. Overall, the estimated correlation coefficients ranged from 0.148 and −0.075, indicating that the association between these six variables and the SWB score was rather low (Table 6).

Following the correlation analyses, the variables that were found to be significantly (*p*-value of <0.05) associated with the SWB score (age, sex, smoking, employment status, marital status, pathology, hours of sleep at night, time spent with friends, time spent with family, 14-MEDAS score or adherence to the MD, time spent in nature, leisure activity, daily basal physical activity, and sport practicing) were all entered into the multiple linear regression model. The data analysis resulted in several good working models, with the best model (Anova *p*-value < 0.000) including all the above independent variables. As shown in Table 7, the model confirmed that several Mediterranean lifestyle characteristics act as positive predictors of the 9-item SWB score; namely, a higher adherence to MD (14-MEDAS score), adequate rest (sleeping longer at night), being active (higher daily normal activity, leisure activity and sport practicing), spending more time socializing with family and friends, and spending more time in nature. Not smoking also acts as positive predictor. Regarding sociodemographic and health related variables, older age, being a man, being married or in an analogous relationship, being employed, and not having a pathology also positively contributes to a higher SWB score. Overall, the combination of these variables explained around 17% of the variance (R = 0.424, R^2^ = 0.180, R^2^ adjusted = 0.173).

## 4. Discussion

The development and application of different tools and terminology to measure well-being perception is partially a consequence of the complexity of fully defining this perception and of establishing all the main components and factors that influence it. Throughout the literature in the area, there appears to be a consensus that multiple-item instruments measuring SWB should include two main dimensions, i.e., hedonic and eudemonic dimensions [5,7,17,43,44,45].

In this study, we have implemented a 9-item tool consisting of a combination of hedonic and eudaimonic items to measure and compare SWB across different populations from MED and non-MED countries. Our results show that, on average, the estimated SWB score was slightly higher in the MED group of participants (6.3) than in the non-MED ones (6.1). The specific individual country scores were: SP = 6.6, PT = 6.2, IT = 6.1, NMK = 6.2, BG = 6.0 (data obtained between 2019 and the start of 2020). Of the two components that are part of the 9-item SWB score, it is interesting to note that small but significant differences between MED and non-MED participants were only achieved for the component containing items related to positive perceptions (7.0 and 6.5, respectively). This difference might be particularly attributed to the lowest scoring for specific questions such as those referring to ‘worthwhile life’ and ‘life satisfaction’, where the difference between participants from BG and the other participants was above one point (data in Supplementary Table S3). Nevertheless, these results are in line with previous cross-cultural studies that reported low overall happiness across the Balkans and Eastern European Post-Communist countries with prevailing Christian Orthodox populations, with the exception of NMK, where the percentage of “very happy” people was close to that in SP and PT [46].

In a reasonable agreement with our results, the life satisfaction by country reported in the most recent WHR (2017–2019) was also ranked higher in the MED countries, SP ≈ IT = 6.4 > PT = 5.9 than in NMK = 5.2 and BG = 5.1 [47]. Similarly, the European Quality of Life Survey (2016) indicated that BG and NMK showed lower life satisfaction values (5.6 and 5.1, respectively) than SP, PT and IT (7.0, 6.9 and 6.6, respectively) [48], and data from the OECD (2018) established a similar ranking for MED countries (SP 7.7, IT 7.4, and PT 7.2) [49]. All these results point to some common issues:

(1) A general moderate perception of SWB across European MED and non-MED countries, in agreement with the ranks reported in Eurostat regarding life satisfaction in 2018 (i.e., 58.6% of Europeans were classified into medium life satisfaction, 25% into high life satisfaction, and 16.4% reported low life satisfaction) [50];
(2) There is a slightly higher SWB perception in participants from the MED countries, than in those of the non-MED countries, with Spanish participants displaying the highest position in the ranking. These results do not appear to have been much altered in recent years;
(3) The measured differences in the SWB score between the countries are rather small, and thus confirming the exact ranking for each country is not a trivial task.

The 9-item SWB tool implemented in this study has supported all these outcomes and may constitute a valuable tool to measure SWB perception in larger populations across different countries. In addition to having a good SWB measuring tool and, given the increasing interest in understanding the relevance of SWB both at the individual and at the society levels, it also remains crucial to clearly delineate the factors that influence well-being perception. These factors could be used as predictors to develop and apply appropriate strategies to improve SWB and its associated co-benefits [13]. Using correlation analyses and different regression models, a considerable number of studies have already provided some evidence of the positive or negative association between SWB and a range of health-related factors, sociodemographic characteristics and lifestyle factors, including age, sex, BMI, education, income or dietary habits. For example, the young to middle-age class, as well as people with tertiary education level, tended to report higher SWB than upper secondary education levels or older age classes [49]. Sex has been found to influence SWB perception, but it was moderated by age [51]. Improved health has also been associated with better SWB, with this association being stronger in developing countries than in the developed ones [52]. In addition, a lower income was associated with higher psychological distress [53], but the association of income inequality and SWB may be influenced by the development of the country economy [54]. In addition, healthy lifestyles have been associated with SWB in SP, where eating specific foods (fruits and vegetables), being in the company of family and friends, engaging in more exercise and feeling healthy have all a positive influence on life satisfaction and happiness [26]. A similar result was reported in IT, where higher adherence to MD or consumption of a vegetable-based dietary pattern were positively associated with psychological resilience [55]. BMI and body image have also been indicated to have a key role for happiness in Spanish adults [56]. In an additional latest study, the consumption of fruits and vegetables has been found associated with life satisfaction in Spanish adults with a high BMI [25]. Overall, the results are still limited, are associated with specific populations, and show some ambiguity, which suggests that these factors may all interact in a complex way to influence SWB.

In the present study, we have implemented correlation analyses and multiple linear regression models between the 9-item SWB score and a broad range of sociodemographic, health related factors, lifestyle and dietary factors in the global sample population from five MED and non-MED countries. Our results further support that closer adherence to MD (measured by 14-MEDAS score) and other Mediterranean lifestyle habits (spending time with friends and family, spending time at nature, leisure activities and practicing sport, and adequate rest at night) have a positive influence on the 9-item SWB score. The association between well-being and a healthy diet has been widely recognized but the specific link of SWB and the consumption of a particular diet or food component is not yet understood. Recently, the lack of evidence for the association between the MD global indicators and SWB has been reported; however, there appears to be some links between the intake of specific food items (fruits, vegetables, sweets) and SWB [25,26]. The complexity of the interaction between diet and SWB deserves further and thorough investigation.

In addition, our results suggest that women seem to have a lower SWB perception, which agrees with data from 29 countries from the European Social Survey, confirming that women are more likely than men to experience depressive symptoms [14]. Our results also point to the fact that having a diagnosed pathology and smoking have a negative influence on the 9-item SWB score, whereas being older has a positive effect. Importantly, our results show that all the variables investigated here display significant but weak correlation coefficients with the 9-item SWB score confirming that our well-being perception is, in part, the result of a multiple factorial influence with small co-effects. Other variables not included here (i.e., income) may contribute to better predicting the 9-item SWB score but, we cannot discard the possibility that other different and more complex models of interaction may occur. Indeed, the association between SWB and all these different factors has been described as a two-direction relationship so that a better SWB can also influence other aspects in our lives [13,57]. Further studies are still needed to clarify the association between the different factors and SWB using a validated instrument applicable to different countries and, if possible, at different time periods. The proposed 9-item SWB score could be a relevant tool for this purpose.

Although there is ample research supporting current self-report measures of SWB with strong psychometric properties with regards to consistency, reliability and validity, there is still skepticism on using self-reported methods to record the construct of SWB since these may be subjected to response bias [5,58]. This relates to whether responders have the willingness to provide honest and adequate answers to the researchers or even if they deceive themselves about their own well-being when responding to the questionnaires [57,58]. In the present study, provision of an online questionnaire might have biased the sample towards a more homogeneous population of middle-class, young to middle aged, with a better education and job status. Indeed, the age distribution of the studied sample was more representative of the working age adult population in Europe, whereas ≥65 years of age respondents constituted only 3% of the participants, and over 70% of participants had a university degree. In addition, from overall 65% employed respondents, more than 90% have a white-collar type of job which suggests that most of these participants have a middle to higher income. It should be reiterated that most studies exploring relationships between SWB, lifestyle and/or sociodemographic factors have a cross-sectional design and can only establish associations but do not permit to draw causality.

Among the strengths of this study is the fact that we assessed a relatively large sample population (N = 2400) including participants from five culturally diverse European countries who provided extensive information regarding various aspects of their well-being and health behavior-related data. Another strength of the study is the restricted eligibility of participants to only native citizens of the respective countries, and the exclusion of participants from other nationalities or those living abroad, in order to minimize the effect of culturally-related factors and/or possible language barriers that may introduce biases when measuring SWB. An important advantage of this study is the assessment of both hedonic and eudemonic indicators, and its combination into a pooled 9-item score which gives us the ability to explore the relationships between subjective dimensions of well-being and sociodemographic, health related factors and lifestyle habits in the different countries. In addition, the application of a finer response scale may give the ability to obtain more detailed insight into a person’s feelings [59], which is implemented in our research by the use of an 11-point Likert-type scale for assessment of every question in the questionnaire.

## 5. Conclusions

Over the past two decades we have outgrown the traditional measures of Gross Domestic Product (GDP) and economic growth as crude assessment tools of the living standards in society. There is a rising interest from governments and policymakers worldwide on the self-reported happiness of individuals. SWB is an essential individual aspect which can impact the development and maintenance of healthy societies. The current study focused on the application of a 9-item SWB tool that includes hedonic and eudemonic indicators, with two components, one that reflects feeling satisfied and fulfilled about life and yourself; and other that reflects aspects of negative psychological functioning, such as high perception of stress, depression, and a sense of being unable to cope with daily tasks. In response to the challenge of a global need to increase SWB, these two domains allow for the identification of issues that should be addressed in policy making. It is clear that in order to influence global well-being, greater emphasis needs to be placed on the individual and, as pointed by some authors, there is an urgent need to promote educational programs in the domains of human flourishing [60]. We have observed significant but rather small differences in SWB among the countries involved in our study, which would need assessment and further confirmation in larger population samples. Furthermore, our results showed significant but small correlations between SWB and several demographic and lifestyle factors (age, sex, nationality, socializing with friends, enjoying nature, sleeping hours, smoking habits, and Mediterranean Diet), highlighting that our well-being perception is the result of a multiple factorial influence. Future research should emphasize the development of improved assessment with accurate instruments that can capture true differences between populations, together with larger scale observational and intervention trials, and comprehensive and functional plans to monitor progress.

## Figures and Tables

**Table 1:** Participants’ sociodemographic characteristics and health status related factors: total population, and MED and non-MED groups of countries.

	Total	MED	Non-MED	*p*-Value ^1^
N (%)	2400	1474	926	
Sex				
Men (%)	801 (33.3)	535 (36.3)	265 (28.6)	0.000
Women (%)	1588 (66.6)	937 (63.7)	661 (71.4)	
Age				
Median (IQR)	38.0 (24.0)	41.0 (26.0)	35.0 (22.0)	
Mean ± SD	38.9 ± 14.1	40.1 ± 14.2	36.7 ± 13.7	0.000
Marital status N (%)				
Single	934 (39.2)	585 (39.8)	349 (38.3)	0.012
Married or analogous relationship	1231 (51.7)	738 (50.2)	493 (54.0)	
Divorced or separated	190 (8.0)	134 (9.1)	56 (6.1)	
Widowed	26 (1.1)	12 (0.8)	14 (1.5)	
Education level N (%)				
Middle school	45.0 (1.9)	43.0 (2.9)	2 (0.2)	0.000
High school	605 (25.3)	259 (17.6)	346 (37.8)	
University degree	959 (40.2)	751 (51.0)	208 (22.8)	
Master’s degree	524 (21.9)	246 (16.7)	278 (30.3)	
Ph.D.	255 (10.7)	174 (11.8)	81 (8.8)	
Employment status N (%)				
Student	507 (21.2)	286 (19.5)	221 (24.1)	0.004
Employed	1555 (65.1)	958 (65.2)	597 (64.8)	
Unemployed part of the year	94 (3.9)	70 (4.8)	24 (2.6)	
Unemployed	150 (6.3)	102 (6.9)	48 (5.2)	
Pensioner (retired, disability)	84 (3.5)	53 (3.6)	31 (3.4)	
BMI (kg/m^2^)				
Median (IQR)	23.70 (5.30)	23.70 (4.90)	23.70 (6.30)	0.789
Mean ± SD	24.41 ± 4.55	24.33 ± 4.21	24.55 ± 5.05	
Disease status N (%)				
Non diagnosed pathology	1842 (79.0)	1079 (75.2)	763 (84.9)	0.000
Diagnosed pathology	491 (21.0)	355 (24.8)	136 (15.1)	

MED: Mediterranean participants from Spain, Italy, and Portugal; non-MED: non-Mediterranean participants from Bulgaria and Republic of North Macedonia; N = Sample size; IQR = interquartile range; SD = standard deviation. N is not constant due to missing data in different variables. ^1^ Mann–Whitney tests were used to assess differences between MED and non-MED participants, for scale variables; Chi-squared tests were used for nominal and ordinal variables.

**Table 2:** Participants’ lifestyle habits: total population, MED and non-MED participants.

	Total	MED	Non-MED	*p*-Value
Smoking N (%)				
Non-smoking	1847 (77.4)	1227 (83.4)	620 (67.9)	0.000
Smoker	538 (22.6)	245 (16.6)	293 (32.1)	
Sleeping hours per night N (%)				
<6 h	431 (18.0)	267 (18.1)	164 (17.9)	0.000
From 6 to 7 h	1107 (46.4)	708 (48.1)	399 (43.0)	
From 7 to 8 h	668 (28.0)	430 (29.2)	238 (26.1)	
From 8 to 10 h	163 (6.9)	64 (4.3)	99 (10.9)	
>10 h	17 (0.7)	3 (0.2)	14 (1.5)	
Do you sleep ‘siesta’? N (%)				
No	1250 (52.1)	741 (50.3)	509 (55.1)	0.005
Yes, but only occasionally	873 (36.4)	541 (36.7)	332 (36.0)	
Yes, frequently	273 (11.4)	190 (13.0)	83 (9.0)	
Time spent in contact with nature N (%)				
Never	234 (9.8)	128 (8.7)	106 (11.6)	0.000
Occasionally	650 (27.4)	456 (31.1)	194 (21.4)	
Sometimes	924 (38.9)	517 (35.2)	407 (44.7)	
Frequently	496 (20.9)	325 (22.2)	171 (18.8)	
Almost all the time	72 (3.0)	40 (2.7)	32 (3.5)	
Time spent with family N (%)				
Never	79 (3.3)	39 (2.7)	40 (4.5)	0.000
Occasionally	321 (13.6)	172 (11.7)	149 (16.6)	
Sometimes	617 (26.2)	384 (26.3)	233 (26.0)	
Frequently	872 (37.0)	598 (40.8)	274 (30.7)	
Almost all the time	468 (19.9)	271 (18.5)	197 (22.1)	
Time spent with friends N (%)				
Never	107 (4.6)	62 (4.2)	45 (5.1)	0.292
Occasionally	508 (21.6)	305 (20.8)	203 (22.9)	
Sometimes	972 (41.5)	605 (41.4)	367 (41.5)	
Frequently	636 (27.1)	416 (28.2)	220 (24.8)	
Almost all the time	123 (5.3)	74 (5.1)	49 (5.6)	
Daily normal activity N (%)				
Normally sat down, don’t walk very much	1082 (45.7)	595 (40.6)	487 (53.9)	0.000
Sometime walking, don’t do strenuous effort	944 (39.8)	629 (43.0)	315 (34.7)	
A lot of time walking, frequent strenuous effort	281 (11.9)	199 (13.7)	82 (9.0)	
A lot of strenuous effort, hard work activity	61 (2.6)	40 (2.7)	21 (2.3)	
Leisure activity N (%)				
Activities that do not require physical activity	782 (33.0)	449 (30.5)	333 (37.0)	0.000
Relaxing activities sometimes per week	1085 (45.7)	621 (42.2)	464 (51.4)	
Sport or intense physical activity	505 (21.3)	400 (27.3)	105 (11.6)	
Sport practicing N (%)				
Never	580 (25.6)	358 (26.7)	222 (24.2)	0.000
Occasionally	793 (35.1)	345 (25.7)	448 (49.0)	
Regularly (<150 min per week)	452 (20.0)	253 (18.8)	199 (21.7)	
Regularly (≥150 min per week)	434 (19.2)	386 (28.8)	48 (5.2)	
Who do you share mean meals with? N (%)				
Alone	461 (19.3)	238 (16.2)	223 (24.2)	0.000
With family or friends	1931 (80.7)	1234 (83.8)	697 (75.8)	
Number of meals per day N (%)				
≤Two	327 (13.8)	81 (5.5)	246 (27.3)	0.000
Three	888 (37.4)	473 (32.1)	415 (46.2)	
Four	642 (27.1)	479 (32.5)	163 (18.2)	
Five	434 (18.3)	376 (25.6)	58 (6.4)	
≥Six	80 (3.4)	63 (4.3)	17 (1.9)	
14-MEDAS score ^1^				
Median (IQR)	7.0 (3.0)	7.00 (3.00)	5.00 (3.00)	0.000
Mean ± SD	6.56 ± 2.13	7.34 ± 1.85	5.57 ± 1.82	

MED: Mediterranean participants from Spain, Italy, and Portugal; non-MED: non-Mediterranean participants from Bulgaria and Republic of North Macedonia; N = Sample size; IQR = interquartile range; SD = standard deviation. N is not constant due to missing data in different variables. For scale variables, Mann–Whitney tests were applied to assess differences between MED and non-MED groups. Chi-squared tests were applied for nominal and ordinal variables; ^1^: 14-MEDAS data are from a previous publication [30].

**Table 3:** Scoring for the individual hedonic and eudemonic items: total population, MED and non-MED participants.

Items (0 = Not at All to 10 = Completely/All the Time)	Total	MED	Non-MED	*p*-Value	SMD
**Eudaimonic Items**					
“Overall, to what extent do you feel that the things you do in your life are worthwhile?”	8.00 (2.00) 7.66 ± 1.75 (2394)	8.00 (2.00) 7.84 ± 1.46 (1473)	8.00 (3.00) 7.36 ± 2.11 (921)	0.000	0.27
“Last week, how efficient did you normally feel in the middle of the day?”	7.00 (3.00) 6.50 ± 1.92 (2393)	7.00 (2.00) 6.63 ± 1.75 (1473)	6.00 (3.00) 6.28 ± 2.16 (920)	0.000	0.18
“Last week, how energetic did you normally feel in the middle of the day?”	6.00 (3.00) 6.20 ± 2.00 (2387)	7.00 (3.00) 6.33 ± 1.80 (1470)	6.00 (3.00) 6.00 ± 2.27 (917)	0.000	0.17
“During last week, how often did you feel confident about your ability to handle your personal problems?”	7.00 (2.00) 6.87 ± 2.22 (2380)	7.00 (2.00) 6.95 ± 2.04 (1470)	7.00 (4.00) 6.76 ± 2.48 (910)	0.477	0.09
“During last week, how often did you feel that you were unable to cope with all the things you had to do?”	4.00 (5.00) 4.28 ± 2.88 (2343)	4.00 (5.00) 4.35 ± 2.83 (1471)	4.00 (4.00) 4.18 ± 2.97 (872)	0.104	0.06
**Hedonic items**					
“Overall, how satisfied are you with your life as a whole these days?”	7.00 (2.00) 7.11 ± 1.97 (2377)	8.00 (1.00) 7.48 ± 1.50 (1473)	7.00 (3.00) 6.50 ± 2.44 (904)	0.000	0.50
“How happy did you feel during the last week?”	7.00 (3.00) 6.81 ± 2.09 (2388)	7.00 (2.00) 6.95 ± 1.86 (1473)	7.00 (3.00) 6.59 ± 2.40 (915)	0.002	0.17
“How worried did you feel during the last week?”	6.00 (5.00) 5.56 ± 2.68 (2364)	6.00 (4.00) 5.74 ± 2.54 (1472)	5.00 (5.00) 5.26 ± 2.86 (892)	0.000	0.18
“Did you feel depressed during the last week?”	2.00 (4.00) 3.24 ± 2.88 (2325)	2.00 (4.00) 3.17 ± 2.76 (1469)	3.00 (6.00) 3.36 ± 3.07 (856)	0.789	−0.07
“Last week, how tired did you normally feel in the middle of the day?”	5.00 (4.00) 5.13 ± 2.41 (2368)	5.00 (4.00) 5.11 ± 2.28 (1472)	5.00 (4.00) 5.17 ± 2.60 (896)	0.938	−0.02
“During last week, how often did you feel nervous and stressed?”	5.00 (4.00) 5.14 ± 2.73 (2380)	6.00 (4.00) 5.21 ± 2.67 (1472)	5.00 (4.00) 5.04 ± 2.83 (908)	0.087	0.06

MED: Mediterranean participants from Spain, Italy, and Portugal; non-MED: non-Mediterranean participants from Bulgaria and Republic of North Macedonia; N = Sample size; IQR = interquartile range; SD = standard deviation. N is not constant due to missing data in different variables. Mann–Whitney tests were applied to assess differences between MED and non-MED groups. SMD: Standardized mean difference (mean score MED group—mean score non-MED group/total SD).

**Table 4:** Results of the exploratory factorial analysis, internal consistency, and convergent validity of the pooled SWB 9-item tool.

	SWB 9-Item Tool	C1 Loadings ^1^	C2 Loadings ^1^
SWB items	Life worthwhile	0.78	
	Efficient	0.75	
	Life satisfaction	0.73	
	Energetic	0.73	
	Feeling happy	0.64	
	Feeling nervous and stressed		0.85
	Feeling worried		0.81
	Unable to cope		0.75
	Feeling depressed		0.68
% of total variance	61.8	31.7	30.1
Eigenvalues ^2^		4.007	1.553
Model adequacy ^3^: KMO/ Bartlett’s	0.83/0.000		
Cronbach’s Alpha ^4^	0.83	0.81	0.81
Composite Reliability ^5^		0.85	0.86
Convergent Validity ^6^		0.54	0.61

^1^ C1 = Component 1; C2 = Component 2; component loadings are the correlation coefficients between each item and the component. ^2^ Only components with eigenvalues above 1 were extracted. ^3^ Good adequacy is considered for KMO values 0.81 to 0.9 and significant Bartlett’s Test of Sphericity (*p* < 0.001). ^4^ Good internal consistency is considered for Cronbach’s Alpha values 0.81 to 0.9. ^5^ Composite Reliability is considered good for values >0.7. ^6^ Convergent Validity is considered acceptable for values >0.5 [41].

**Table 5:** Pooled 9-item score and components C1 and C2: total population, MED and non-MED participants.

Scale 0 = Not at All to 10 = Completely/All the Time	Total	MED	Non-MED	*p*-Value	SMD
Pooled 9-item score ^1^					
Median (IQR)	6.3 (2.1)	6.4 (2.1)	6.1 (2.3)		0.13
Mean ± SD	6.2 ± 1.5	6.3 ± 1.5	6.1 ± 1.6		
N (% of respondents) ^2^	2261 (94.2%)	1465 (99.4%)	796 (86.0%)	0.002	
C1 (positive perceptions) ^3^					
Median (IQR)	7.0 (1.8)	7.2 (1.4)	6.8 (2.4)		0.26
Mean ± SD	6.9 ± 1.5	7.0 ± 1.3	6.5 ± 1.6		
N (% of respondents)	2356 (97.9%)	1470 (99.7%)	886 (95.7%)	0.000	
C2 (negative perceptions) ^4^					
Median (IQR)	4.5 (4.0)	4.7 (3.5)	4.5 (3.5)		0.04
Mean ± SD	4.6 ± 2.2	4.6 ± 2.2	4.5 ± 2.3		
N (% of respondents)	2289 (95.1%)	1466 (99.5%)	823 (88.9%)	0.115	

MED: Mediterranean participants from Spain, Italy, and Portugal; non-MED: non-Mediterranean participants from Bulgaria and Republic of North Macedonia. N = Sample size; IQR = interquartile range; SD = standard deviation. N is not constant due to missing data in different variables. Mann–Whitney tests were applied to assess differences between MED and non-MED groups. SMD: Standardized mean difference (mean score MED group—mean score non-MED group/total SD). ^1^ The pooled 9-item score is obtained by calculating the mean of the C1 items and inverted C2 items. ^2^ Percentage of respondents refers to the percentage of participants that answered all the questions necessary for the calculation of the pooled 9-item score, and components. ^3^ C1 includes the items related to positive perceptions of SWB: life satisfaction, worthwhile life, feeling happy, energetic, and efficient. ^4^ C2 includes the items related to negative perceptions: feeling worried, feeling depressed, feeling nervous and stressed, and being unable to cope.

**Table 6:** Non-parametric partial correlations between sociodemographic, health status related factors, and MED lifestyle factors and the 9-item SWB score.

Variables ^1^	Correlation Coefficient (*ρ*)	*p*-Value
Sociodemographic		
Age	**0.107**	**0.000**
Sex (ref = men)	**−0.107**	**0.000**
Employment status (ref = full time job)	−0.093	**0.000**
Marital status (ref = single)	0.051	0.032
Education level	0.010	0.682
Household	0.023	0.343
Lifestyle		
Smoking (ref = no smoking)	−0.047	0.047
Rest		
Sleeping hours at night	**0.148**	**0.000**
Sleeping ‘Siesta’	−0.018	0.445
Physical activity		
Daily normal activity	0.062	0.009
Leisure activity	0.057	0.016
Sport practicing	0.057	0.017
Food habits		
MD adherence	0.067	0.005
Meals per day	0.003	0.889
Social habits		
Sharing meals	0.035	0.143
Time spent with family	0.058	0.015
Time spent with friends	**0.105**	**0.000**
Time spent in nature	0.070	0.003
Health status		
Pathology (ref = no pathology)	−0.075	**0.002**
BMI	−0.005	0.835

N = 2400; Nationality used as control variable. Significant correlation below Bonferroni cut-off (*p* < 0.0025) are in bold. Correlation factor above 0.1 are also in bold. MD adherence was assessed by the 14-MEDAS. The pooled 9-item score is obtained by calculating the mean of: life satisfaction, worthwhile life, feeling happy, energetic, and efficient, and inverted items: feeling worried, feeling depressed, feeling nervous and stressed, and being unable to cope. ^1^ In nominal variables the correlations are presented are related to the reference: women vs. men; full time job vs. student, unemployed or pensioner; single vs. married or analogous relationship, divorced or separated, widowed; non-smokers vs. smokers; no pathology vs. presence of pathology.

**Table 7:** Multiple linear regression model to assess the relationship between the pooled 9-item SWB score and sociodemographic, health related and lifestyle factors.

	9-Item SWB Score			
Variable ^1^	T	SE	β	*p*-Value
Age	0.013	0.003	0.113	0.000
Sex	−0.324	0.070	−0.101	0.000
Marital status	0.237	0.074	0.078	0.002
Employment status	−0.375	0.078	−0.116	0.000
Pathology	−0.289	0.082	−0.078	0.000
Smoking	−0.208	0.081	−0.056	0.010
Sleeping hours at night	0.252	0.040	0.140	0.000
MD adherence	0.061	0.018	0.081	0.001
Daily normal activity	0.097	0.043	0.049	0.025
Leisure activity	0.151	0.059	0.074	0.010
Sport practicing	0.094	0.040	0.067	0.019
Time spent with friends	0.198	0.039	0.120	0.000
Time spent with family	0.104	0.034	0.071	0.002
Time spent in nature	0.141	0.036	0.089	0.000

T = Student *t* test; SE = Standard Error; β = regression coefficient. Anova *p*-value < 0.000, R = 0.424, R^2^ = 0.180, R^2^ adjusted = 0.173. MD adherence was assessed by the 14-MEDAS. The pooled 9-item score is obtained by calculating the mean of: life satisfaction, worthwhile life, feeling happy, energetic, and efficient, and inverted items: feeling worried, feeling depressed, feeling nervous and stressed, and being unable to cope. ^1^ Reference to the nominal variables present in the final model are: sex- men; employment status—full-time job; marital status—single; smoking—non-smokers; pathology—no pathology.

## Data Availability

Results attained in this study are included in the manuscript and in the Supplementary Materials. Individual data are not publicly available due to ethical restrictions.

## Supplementary Materials

The following supporting information can be downloaded at: https://www.mdpi.com/article/10.3390/ijerph19031715/s1, Table S1: Participants’ demographic and socioeconomic characteristics: global population, individual countries, and MED and non-MED regions. Table S2: Participants’ lifestyle: global population, individual countries, and MED and non-MED regions. Table S3: Scoring for the individual hedonic and eudemonic items: total population, individual countries, and MED and non-MED groups. Table S4: Summary of factor analysis per country. Table S5 Comparison of the pooled 9-item SWB score, and components C1 and C2 among countries, and MED and non-MED participants.
