# Supplementary material for: Exploring Hedonic and Eudaimonic Items of Well-Being in Mediterranean and Non-Mediterranean Countries: Influence of Sociodemographic and Lifestyle Factors
Source: Int J Environ Res Public Health. 2022 Feb 2;19(3):1715. doi: 10.3390/ijerph19031715 (PMC8835089; doi:10.3390/ijerph19031715)
Supplement: Supplementary file 1 [file ijerph-19-01715-s001.zip › ijerph-1546669-supplementary.pdf]

Table S1. Participants' sociodemographic characteristics and health related factors: total population, individual countries, and MED and non-MED groups.

	Total	SP	IT	PT[1]	MED	BG	NMK	Non-MED	*p*-value[2]	*p*-value[3]
N (%)	2400	485 (20.1)	505 (21.0)	484 (20.2)	1474	492 (20.5)	434 (18.1)	926		
Sex										
Men (%)	801 (33.3)	209 (28.0)	190 (37.6)	136 (28.2)	535 (36.3)	152 (30.9)	113 (26.0)	265 (28.6)	0.000	0.000
Women (%)	1588 (66.6)	276 (71.5)	315 (62.4)	346 (71.8)	937 (63.7)	340 (69.1)	321 (73.9)	661 (71.4)		
Age										
Median (IQR)	38.0 (24.0)	49.0 (18.0)	36.0 (25.0)	34.0 (24.0)	41.0 (26.0)	42.0 (16.7)	24.0 (14.0)	35.0 (22.0)		
Mean ± SD	38.9 ± 14.1	45.7 ± 12.8	38.2 ± 14.2	36.5 ± 13.7	40.1 ± 14.2	43.0 ± 12.7	29.5 ± 10.9	36.7 ± 13.7	0.000	0.000
Marital status N (%)										
Single	934 (39.2)	137 (28.5)	222 (44.0)	226 (46.7)	585 (39.8)	109 (22.3)	240 (56.6)	349 (38.3)	0.000	0.012
Married or analogous relationship	1231 (51.7)	262 (54.6)	257 (50.9)	219 (45.2)	738 (50.2)	315 (64.5)	178 (42.0)	493 (54.0)		
Divorced or separated	190 (8.0)	76 (15.8)	23 (4.6)	35 (7.2)	134 (9.1)	52 (10.7)	4 (0.9)	56 (6.1)		
Widowed	26 (1.1)	5 (1.0)	3 (0.6)	4 (0.8)	12 (0.8)	12 (2.5)	2 (0.5)	14 (1.5)		
Education level N (%)										
Middle school	45.0 (1.9)	15 (3.1)	12 (2.4)	16.0 (3.3)	43.0 (2.9)	2 (0.4)	0 (0.0)	2 (0.2)	0.000	0.000
High school	605 (25.3)	71 (14.7)	59 (11.7)	129 (26.7)	259 (17.6)	93 (19.1)	253 (59.0)	346 (37.8)		
University degree	959 (40.2)	184 (38.0)	358 (70.9)	209 (43.2)	751 (51.0)	90 (18.5)	118 (27.7)	208 (22.8)		
Master degree	524 (21.9)	93 (19.2)	53 (10.5)	100 (20.7)	246 (16.7)	236 (48.5)	42 (9.8)	278 (30.3)		
Ph.D.	255 (10.7)	121 (25.0)	23 (4.6)	30 (6.2)	174 (11.8)	66 (13.6)	15 (3.5)	81 (8.8)		
Employment status N (%)										
Student	507 (21.2)	44 (9.1)	136 (27.1)	106 (21.9)	286 (19.5)	19 (3.9)	202 (46.6)	221 (24.1)	0.000	0.004
Employed	1555 (65.1)	380 (78.7)	241 (48.0)	337 (69.6)	958 (65.2)	392 (80.5)	205 (47.2)	597 (64.8)		
Unemployed part of the year	94 (3.9)	18 (3.7)	38 (7.6)	14 (2.9)	70 (4.8)	19 (3.9)	5 (1.1)	24 (2.6)		
Unemployed	150 (6.3)	24 (5.0)	64 (12.7)	14 (2.9)	102 (6.9)	30 (6.2)	18 (4.1)	48 (5.2)		
Pensioner (retired, disability)	84 (3.5)	17 (3.5)	23 (4.6)	13 (2.7)	53 (3.6)	27 (5.5)	4 (0.9)	31 (3.4)		
Type of employment N (%)										
White collar	1385 (90.1)	360 (90.9)	257 (78.8)	321 (97.9)	938 (89.3)	260 (90.6)	187 (93.0)	447 (91.6)	**0.000**	0.167
Blue collar	153 (9.9)	36.0 (9.1)	69.0 (21.2)	7.0 (2.1)	112 (10.7)	27.0 (9.4)	14 (7.0)	41.0 (8.4)		
BMI[1]										
Median (IQR)	23.70 (5.30)	24.20 (4.40)	23.50 (4.80)	23.40 (5.20)	23.70 (4.90)	24.20 (6.70)	23.30 (5.20)	23.70 (6.30)	0.000	0.789
Mean ± SD	24.41 ± 4.55	24.65 ± 3.93	24.13 ± 4.10	24.20 ± 4.56	24.33 ± 4.21	25.23 ± 5.66	23.76 ± 4.08	24.55 ± 5.05		
Disease status N (%)										
Non diagnosed pathology	1842 (79.0)	317 (66.7)	423 (83.8)	339 (74.7)	1079 (75.2)	401 (83.9)	362 (86.0)	763 (84.9)	0.000	0.000
Diagnosed pathology	491 (21.0)	158 (33.3)	82 (16.2)	115 (23.8)	355 (24.8)	77 (16.1)	59 (14.0)	136 (15.1)		

SP: Spain; IT: Italy; PT: Portugal; BG: Bulgaria; NMK: Republic of North Macedonia; MED: Mediterranean; non-MED: not Mediterranean; N = Sample size; IQR= interquartile range; SD= standard deviation. N is not constant due to missing data in different variables. Mann-Whitney and Kruskal-Wallis tests were used to assess differences between groups of countries (MED and non-MED) and between individual countries, respectively, for scale variables; Chi-squared tests were used for nominal and ordinal variables; [1]: some of the data for the PT sample population are from a previous publication [29], as well as BMI data [30]; [2]: *p*-values between all individual countries; [3]: *p*-values between MED and non-MED regions.

Table S2. Participants' lifestyle habits: total population, individual countries, and MED and non-MED groups.

	Total	SP	IT	PT[1]	MED	BG	NMK	Non-Med	*p*-value[3]	*p*-value[4]
Smoking N (%)										
Non-smoking	1847 (77.4)	415 (85.8)	418 (82.8)	394 (81.4)	1227 (83.4)	313 (64.9)	307 (71.1)	620 (67.9)	0.000	0.000
Smoker	538 (22.6)	69 (14.2)	86 (17.0)	90 (18.6)	245 (16.6)	169 (35.1)	124 (28.9)	293 (32.1)		
Sleeping hours per night N (%)										
< 6 h	431 (18.0)	98 (20.2)	98 (19.4)	71 (14.7)	267 (18.1)	92 (18.8)	72 (16.9)	164 (17.9)	0.000	0.000
From 6 to 7 h	1107 (46.4)	238 (49.3)	252 (49.9)	218 (45.0)	708 (48.1)	210 (42.9)	189 (44.4)	399 (43.0)		
From 7 to 8 h	668 (28.0)	127 (26.2)	135 (26.7)	168 (34.7)	430 (29.2)	143 (29.2)	95 (22.5)	238 (26.1)		
From 8 to 10 h	163 (6.9)	21 (4.3)	18 (3.6)	25 (5.2)	64 (4.3)	42 (8.6)	57 (13.6)	99 (10.9)		
> 10 h	17 (0.7)	0 (0)	1 (0.2)	2 (0.4)	3 (0.2)	3 (0.6)	11 (2.6)	14 (1.5)		
Do you sleep 'siesta'? N (%)										
No	1250 (52.1)	191 (39.3)	217 (43.0)	333 (68.8)	741 (50.3)	353 (71.7)	156 (36.2)	509 (55.1)	0.000	0.005
Yes, but only occasionally	873 (36.4)	196 (40.3)	201 (39.8)	144 (29.8)	541 (36.7)	116 (23.6)	216 (50.0)	332 (36.0)		
Yes, frequently	273 (11.4)	98 (20.4)	85 (16.8)	7 (1.4)	190 (13.0)	23 (4.7)	60 (13.8)	83 (9.0)		
Time spent in nature N (%)										
Never	234 (9.8)	35 (7.3)	57 (11.3)	36 (7.4)	128 (8.7)	55 (11.4)	51 (11.9)	106 (11.6)	0.000	0.000
Occasionally	650 (27.4)	167 (34.7)	161 (31.9)	128 (26.4)	456 (31.1)	57 (11.8)	137 (32.2)	194 (21.4)		
Sometimes	924 (38.9)	136 (28.3)	207 (41.0)	174 (36.0)	517 (35.2)	248 (51.2)	159 (37.4)	407 (44.7)		
Frequently	496 (20.9)	129 (27.0)	66 (13.1)	130 (26.9)	325 (22.2)	103 (21.3)	68 (15.9)	171 (18.8)		
Almost all the time	72 (3.0)	13 (2.7)	11 (2.2)	16 (3.3)	40 (2.7)	21 (4.3)	11 (2.6)	32 (3.5)		
Time spent with family N (%)										
Never	79 (3.3)	9 (1.9)	18 (3.6)	12 (2.5)	39 (2.7)	22 (4.6)	18 (4.3)	40 (4.5)	0.000	0.000
Occasionally	321 (13.6)	69 (14.3)	49 (9.7)	54 (11.2)	172 (11.7)	71 (15.0)	78 (18.5)	149 (16.6)		
Sometimes	617 (26.2)	88 (18.5)	193 (38.2)	103 (21.3)	384 (26.3)	130 (27.4)	103 (24.5)	233 (26.0)		
Frequently	872 (37.0)	210 (43.7)	154 (30.5)	234 (48.3)	598 (40.8)	147 (31.0)	127 (30.4)	274 (30.7)		
Almost all the time	468 (19.9)	104 (21.6)	86 (17.0)	81 (16.7)	271 (18.5)	104 (21.9)	93 (22.3)	197 (22.1)		
Time spent with friends N (%)										
Never	107 (4.6)	13 (2.7)	30 (5.9)	19 (3.9)	62 (4.2)	34 (7.4)	11 (2.6)	45 (5.1)	0.000	0.292
Occasionally	508 (21.6)	126 (26.3)	77 (15.2)	102 (21.1)	305 (20.8)	105 (22.8)	98 (23.1)	203 (22.9)		
Sometimes	972 (41.5)	172 (36.0)	241 (47.7)	192 (39.7)	605 (41.4)	208 (45.1)	159 (37.6)	367 (41.5)		
Frequently	636 (27.1)	149 (31.0)	127 (25.1)	140 (28.9)	416 (28.2)	109 (23.6)	111 (26.1)	220 (24.8)		
Almost all the time	123 (5.3)	19 (4.0)	24 (4.8)	31 (6.4)	74 (5.1)	5 (1.1)	44 (10.6)	49 (5.6)		
Daily normal activity N (%)										
Normally sat down, don't walk very much	1082 (45.7)	202 (42.2)	232 (45.9)	161 (33.3)	595 (40.6)	343 (70.3)	144 (34.5)	487 (53.9)	0.000	0.000
Sometime walking, don't do strenuous effort	944 (39.8)	203 (42.4)	209 (41.4)	217 (44.8)	629 (43.0)	104 (21.3)	211 (50.7)	315 (34.7)		
A lot of time walking, frequent strenuous effort	281 (11.9)	68 (14.4)	42 (8.3)	89 (18.4)	199 (13.7)	28 (5.7)	54 (12.9)	82 (9.0)		
A lot of strenuous effort, hard work activity	61 (2.6)	5 (1.0)	18 (3.6)	17 (3.5)	40 (2.7)	13 (2.7)	8 (1.9)	21 (2.3)		
Leisure activity N (%)										
Activities that do not require physical activity	782 (33.0)	83 (17.1)	174 (34.5)	192 (39.7)	449 (30.5)	164 (33.4)	169 (41.1)	333 (37.0)	0.000	0.000
Relaxing activities sometimes per week	1085 (45.7)	246 (50.7)	208 (41.2)	167 (34.5)	621 (42.2)	276 (56.2)	188 (45.8)	464 (51.4)		
Sport or intense physical activity	505 (21.3)	155 (32.2)	120 (23.8)	125 (25.8)	400 (27.3)	51 (10.4)	54 (13.1)	105 (11.6)		
Sport practising N (%)										
Never	580 (25.6)	45 (9.4)	203 (40.2)	110 (22.7)	358 (26.7)	115 (23.4)	107 (25.2)	222 (24.2)	0.000	0.000
Occasionally	793 (35.1)	122 (25.4)	115 (22.8)	108 (22.3)	345 (25.7)	237 (48.2)	211 (49.6)	448 (49.0)		
Regularly (< 150 min per week)	452 (20.0)	114 (23.7)	71 (14.1)	68 (14.0)	253 (18.8)	135 (27.4)	64 (15.1)	199 (21.7)		

Regularly (≥ 150 min per week)	434 (19.2)	199 (41.6)	106 (21.0)	81 (16.7)	386 (28.8)	5 (1.0)	43 (10.1)	48 (5.2)		
Who do you share mean meals with? N (%)										
Alone	461 (19.3)	98 (20.4)	57 (11.3)	83 (17.1)	238 (16.2)	126 (25.6)	97 (22.5)	223 (24.2)	0.000	0.000
With family or friends	1931 (80.7)	387 (79.6)	446 (88.3)	401 (82.9)	1234 (83.8)	363 (73.8)	334 (77.5)	697 (75.8)		
Number of meals per day N (%)										
≤ Two	327 (13.8)	15 (3.1)	48 (9.5)	18 (3.7)	81 (5.5)	102 (21.7)	144 (33.5)	246 (27.3)	0.000	0.000
Three	888 (37.4)	169 (34.8)	204 (40.4)	100 (20.7)	473 (32.1)	221 (46.9)	194 (45.3)	415 (46.2)		
Four	642 (27.1)	178 (36.7)	135 (26.7)	166 (34.7)	479 (32.5)	96 (20.4)	67 (15.8)	163 (18.2)		
Five	434 (18.3)	115 (23.9)	109 (21.6)	152 (31.4)	376 (25.6)	39 (8.3)	19 (4.4)	58 (6.4)		
≥ Six	80 (3.4)	7 (1.4)	8 (1.6)	48 (9.9)	63 (4.3)	13 (2.8)	4 (0.9)	17 (1.9)		
14-MEDAS score[2]										
Median (IQR)	7.0 (3.0)	8.00 (2.00)	7.0 (2.0)	7.0 (3.0)	7.00 (3.00)	6.00 (2.00)	5.00 (2.75)	5.00 (3.00)	0.000	0.000
Mean ± SD	6.56 ± 2.13	7.90 ± 1.72	6.79 ± 1.54	7.37 ± 2.10	7.34 ± 1.85	5.80 ± 1.77	5.30 ± 1.85	5.57 ± 1.82		

SP: Spain; IT: Italy; PT: Portugal; BG: Bulgaria; NMK: Republic of North Macedonia; MED: Mediterranean; non-MED: not Mediterranean; N = Sample size; IQR= interquartile range; SD= standard deviation. N is not constant due to missing data in different variables. For scale variables, Mann-Whitney and Kruskal-Wallis tests were applied to assess differences between groups of countries (MED and non-MED) and between individual countries, respectively. Chi-squared tests were applied for nominal and ordinal variables. [1]: some of the data for the PT sample population are from a previous publication [29]; [2]: 14-MEDAS data are from a previous publication [30]; [3]: *p*-values between all individual countries; [4]: *p*-values between MED and non-MED regions.

Table S3. Scoring for the individual hedonic and eudemonic items: total population, individual countries, and MED and non-MED groups.

Items (scale 0 to 10)	Total	SP	IT	PT	MED	BG	NMK	Non-MED	*p*-value(1)	*p*-value(2)	SMD
Eudemonic items											
"Overall, to what extent do you feel that the things you do in your life are worthwhile?"	8.00 (2.00)<br>7.66 ± 1.75<br>(2394)	8.00 (2.00)<br>7.93 ± 1.38<br>(484)	8.00 (2.00)<br>7.88 ± 1.40<br>(505)	8.00 (2.00)<br>7.73 ± 1.60<br>(484)	8.00 (2.00)<br>7.84 ± 1.46<br>(1473)	7.00 (3.00)<br>6.85 ± 2.05<br>(487)	8.00 (3.00)<br>7.93 ± 2.02<br>(434)	8.00 (3.00)<br>7.36 ± 2.11<br>(921)	0.000	0.000	0.27
"Last week, how efficient did you normally feel in the middle of the day?"	7.00 (3.00)<br>6.50 ± 1.92<br>(2393)	7.00 (2.00)<br>6.76 ± 1.68<br>(484)	7.00 (2.00)<br>6.75 ± 2.74<br>(505)	7.00 (3.00)<br>6.38 ± 1.88<br>(484)	7.00 (2.00)<br>6.63 ± 1.75<br>(1473)	6.00 (3.00)<br>6.13 ± 1.98<br>(487)	7.00 (3.00)<br>6.45 ± 2.34<br>(433)	6.00 (3.00)<br>6.28 ± 2.16<br>(920)	0.000	0.000	0.18
"Last week, how energetic did you normally feel in the middle of the day?"	6.00 (3.00)<br>6.20 ± 2.00<br>(2387)	7.00 (3.00)<br>6.44 ± 1.70<br>(482)	7.00 (2.00)<br>6.60 ± 1.70<br>(504)	6.00 (2.00)<br>5.94 ± 1.92<br>(484)	7.00 (3.00)<br>6.33 ± 1.80<br>(1470)	6.00 (2.00)<br>5.80 ± 2.29<br>(483)	6.00 (3.00)<br>6.21 ± 2.24<br>(434)	6.00 (3.00)<br>6.00 ± 2.27<br>(917)	0.000	0.000	0.17
"During last week, how often did you feel that you were unable to cope with all the things you had to do?"	4.00 (5.00)<br>4.28 ± 2.88<br>(2343)	3.00 (5.00)<br>3.70 ± 2.90<br>(483)	5.00 (4.00)<br>5.19 ± 2.66<br>(504)	4.00 (4.00)<br>4.12 ± 2.72<br>(484)	4.00 (5.00)<br>4.35 ± 2.83<br>(1471)	3.00 (5.00)<br>3.71 ± 2.76<br>(439)	5.00 (5.00)<br>4.66 ± 3.09<br>(433)	4.00 (4.00)<br>4.18 ± 2.97<br>(872)	0.000	0.104	0.06
"During last week, how often did you feel confident about your ability to handle your personal problems?"	7.00 (2.00)<br>6.87 ± 2.22<br>(2380)	8.00 (3.00)<br>7.28 ± 2.02<br>(482)	7.00 (2.00)<br>6.85 ± 1.98<br>(504)	7.00 (3.00)<br>6.72 ± 2.07<br>(484)	7.00 (2.00)<br>6.95 ± 2.04<br>(1470)	7.00 (3.00)<br>6.56 ± 2.55<br>(476)	7.50 (4.00)<br>6.97 ± 2.40<br>(434)	7.00 (4.00)<br>6.76 ± 2.48<br>(910)	0.000	0.477	0.09
Hedonic items											
"Overall, how satisfied are you with your life as a whole these days?"	7.00 (2.00)<br>7.11 ± 1.97<br>(2377)	8.00 (2.00)<br>7.76 ± 1.38<br>(484)	8.00 (1.00)<br>7.44 ± 1.55<br>(505)	7.00 (1.00)<br>7.25 ± 1.53<br>(484)	8.00 (1.00)<br>7.48 ± 1.50<br>(1473)	6.00 (3.00)<br>5.87 ± 2.63<br>(470)	8.00 (3.00)<br>7.18 ± 1.99<br>(434)	7.00 (3.00)<br>6.50 ± 2.44<br>(904)	0.000	0.000	0.50
"How happy did you feel during the last week?"	7.00 (3.00)<br>6.81 ± 2.09<br>(2388)	8.00 (2.00)<br>7.32 ± 1.62<br>(484)	7.00 (2.00)<br>6.68 ± 1.97<br>(505)	7.00 (2.00)<br>6.87 ± 1.93<br>(484)	7.00 (2.00)<br>6.95 ± 1.86<br>(1473)	7.00 (3.00)<br>6.43 ± 2.33<br>(482)	7.00 (4.00)<br>6.76 ± 2.45<br>(433)	7.00 (3.00)<br>6.59 ± 2.40<br>(915)	0.000	0.002	0.17
"How worried did you feel during the last week?"	6.00 (5.00)<br>5.56 ± 2.68<br>(2364)	6.00 (4.00)<br>5.28 ± 2.60<br>(483)	6.00 (4.00)<br>6.09 ± 2.46<br>(505)	6.00 (4.00)<br>5.84 ± 2.51<br>(484)	6.00 (4.00)<br>5.74 ± 2.54<br>(1472)	5.00 (4.00)<br>4.78 ± 2.60<br>(458)	6.00 (5.00)<br>5.75 ± 3.03<br>(434)	5.00 (5.00)<br>5.26 ± 2.86<br>(892)	0.000	0.000	0.18
"Did you feel depressed during the last week?"	2.00 (4.00)<br>3.24 ± 2.88<br>(2325)	2.00 (5.00)<br>2.65 ± 2.76<br>(482)	3.00 (5.00)<br>3.56 ± 2.67<br>(503)	3.00 (4.00)<br>3.28 ± 2.78<br>(484)	2.00 (4.00)<br>3.17 ± 2.76<br>(1469)	2.00 (4.00)<br>3.32 ± 2.98<br>(422)	3.00 (6.00)<br>3.41 ± 3.17<br>(434)	3.00 (6.00)<br>3.36 ± 3.07<br>(856)	0.000	0.789	-0.07
"Last week, how tired did you normally feel in the middle of the day?"	5.00 (4.00)<br>5.13 ± 2.41<br>(2368)	5.00 (4.00)<br>4.95 ± 2.27<br>(483)	5.00 (4.00)<br>5.24 ± 2.18<br>(505)	5.00 (4.00)<br>5.15 ± 2.39<br>(484)	5.00 (4.00)<br>5.11 ± 2.28<br>(1472)	5.00 (3.00)<br>4.69 ± 2.43<br>(464)	6.00 (4.00)<br>5.68 ± 2.67<br>(432)	5.00 (4.00)<br>5.17 ± 2.60<br>(896)	0.000	0.938	-0.02
"During last week, how often did you feel nervous and stressed?"	5.00 (4.00)<br>5.14 ± 2.73<br>(2380)	5.00 (5.00)<br>4.77 ± 2.73<br>(484)	6.00 (400)<br>5.79 ± 2.42<br>(504)	5.00 (4.00)<br>5.03 ± 2.75<br>(484)	6.00 (4.00)<br>5.21 ± 2.67<br>(1472)	5.00 (5.00)<br>4.50 ± 2.61<br>(474)	5.50 (5.00)<br>5.62 ± 2.94<br>(434)	5.00 (4.00)<br>5.04 ± 2.83<br>(908)	0.000	0.087	0.06

MED: Mediterranean; non-MED: Not Mediterranean; SP: Spain; IT: Italy; PT: Portugal; BG: Bulgaria; NMK: Republic of North Macedonia; Values are presented as median (IQR) and mean ± SD; (N): size of sample included in the analysis; (1): The Kruskal-Wallis test was used to assess differences between countries. (2): The Mann-Whitney U test was used to assess differences between the MED group and non-MED group of countries. SMD: Standardized mean difference (mean score MED group – mean score non-MED group/total SD).

Supplementary Table S4. Summary of factor analysis per country

Country (N)	PT (488)		SP (463)		IT (501)		NMK (340)		BG (327)	
Weight of items[1] in extracted factors	C1	C2	C1	C2	C1	C2	C1	C2	C1	C2
Life satisfaction	0.745		0.715		0.704		0.621		0.727	
Feeling Happy	0.740		0.730		0.591		0.580		0.485	
Energetic	0.744		0.775		0.755		0.743		0.612	
Worthwhile life	0.785		0.763		0.753		0.787		0.792	
Efficient	0.768		0.800		0.807		0.776		0.629	
Feeling worried		0.840		0.792		0.829		0.816		0.774
Feeling depressed		0.709		0.650		0.640		0.723		0.653
Nervous and stressed		0.851		0.857		0.858		0.833		0.844
Unable to cope		0.713		0.782		0.734		0.741		0.638
% of total variance (subscales)	34.2	30.0	34.3	30.2	32.3	30.4	29.5	31.9	26.0	28.1
% of total variance (9-item-SWB	64.2		64.5		62.7		61.4		54.1	
KMO/Bartletts [2]	0.841/<0.001		0.829/<0.001		0.829/<0.001		0.859/<0.001		0.771/<0.001	
Chronbach's Alpha (9-item-SWB) [3]	0.855		0.847		0.850		0.846		0.793	
Chronbach's Alpha (components)	0.844	0.823	0.844	0.820	0.815	0.816	0.799	0.820	0.746	0.750
Composite Reliability [4]	0.87	0.86	0.87	0.86	0.85	0.85	0.83	0.86	0.79	0.82
Convergent Validity [5]	0.57	0.61	0.57	0.60	0.53	0.59	0.50	0.61	0.43	0.54

[1] Only components with eigenvalues above 1 were extracted. Items: Life satisfaction - Overall, how satisfied are you with your life as a whole these days?; Feeling happy - How happy did you feel during the last week?; Energetic - Last week, how energetic did you feel in the middle of the day?; Worthwhile life - To what extent do you feel that the things you do in life are worthwhile?; Efficient - Last week, how efficient did you feel in the middle of the day?; Feeling worried - How worried did you feel during the last week?; Feeling depressed - Did you feel depressed during the last week?; Nervous and stressed - During last week, how often did you feel nervous and stressed?; Unable to cope - During last week, how often did you feel that you were unable to cope with all the thing you had to do? All questions were assessed using a 10 point Likert-type scale, (0 = not at all to 10 = completely/all the time). To have all questions coded in the same direction for factor analysis, an inverted scale, 10 - "x", was applied to the following questions: feeling worried; feeling depressed; feeling nervous and stressed; unable to cope.

[2]Good adequacy is considered for KMO values 0.81 to 0.9 and significant Bartlett's Test of Sphericity ($p<0.001$).

[3] Good internal consistency is considered for Cronbach's Alpha values 0.81 to 0.9.

[4] Composite Reliability is considered good for values >0.7.

[5] Convergent Validity is considered acceptable for values >0.5

Table S5. Comparison of the pooled 9-item SWB score, and components C1 and C2 among countries, and MED and non-MED participants.

	**9-item SWB score**<br>**N (% of respondents)**[1]<br>**Median (IQR)**<br>**Mean ± SD**	**C1**<br>**N (% of respondents)**[1]<br>**Median (IQR)**<br>**Mean ± SD**	**C2**<br>**N (% of respondents)**[1]<br>**Median (IQR)**<br>**Mean ± SD**
All countries	2261 (94.2%)<br>6.3 (2.11)<br>6.2 ± 1.5	2356 (97.9%)<br>7.0 (1.80)<br>6.9 ± 1.5	2289 (95.1%)<br>4.5 (4.0)<br>4.6 ± 2.2
SP	479 (98.8%)<br>6.8 (2.0)<br>6.6 ± 1.4	482 (99.4%)<br>7.4 (1.40)<br>7.2 ± 1.2	480 (99.0%)<br>4.0 (3.5)<br>4.1 ± 2.2
PT	484 (100%)<br>6.33 (2.11)<br>6.2 ± 1.5	484 (100%)<br>7.0 (1.80)<br>6.8 ± 1.4	484 (100%)<br>4.75 (3.5)<br>4.5 ± 2.2
IT	502 (99.4%)<br>6.1 (1.89)<br>6.1 ± 1.4	504 (99.8%)<br>7.2 (1.0)<br>7.1 ± 1.3	502 (99.4%)<br>5.2 (3.25)<br>5.2 ± 2.0
BG	365 (74.2%)<br>6.0 (2.33)<br>6.0 ± 1.5	454 (92.3%)<br>6.3 (2.40)<br>6.1 ± 1.6	390 (79.3%)<br>4.0 (3.25)<br>4.2 ± 2.1
NMK	431 (99.3%)<br>6.22 (2.33)<br>6.1 ± 1.7	432 (99.5%)<br>7.2 (2.0)<br>6.9 ± 1.6	433 (99.8%)<br>4.75 (3.75)<br>4.8 ± 2.4
p-value [2]	0.000	0.000	0.000
MED	1465 (99.4%)<br>6.4 (2.11)<br>6.3 ± 1.5	1470 (99.7%)<br>7.2 (1.45)<br>7.0 ± 1.3	1466 (99.5%)<br>4.75 (3.5)<br>4.6 ± 2.2
Non-MED	796 (86.0%)<br>6.1 (2.33)<br>6.1 ± 1.6	886 (95.7%)<br>6.8 (2.4)<br>6.5 ± 1.6	823 (88.9%)<br>4.5 (3.5)<br>4.5 ± 2.3
p-value [3]	0.002	0.000	0.115

[1] N = Sample size (N is not constant due to missing data in different variables); percentage of respondents refers to the percentage of participants that answered all the questions necessary for the calculation of 9-item SWB Index, C1, and C2 components, respectively. Scales 9-item SWB index, S&M and D&S were scored in a 10 point Likert-type scale from 0 (lowest) to 10 (highest). SP: Spain; IT: Italy; PT: Portugal; BG: Bulgaria; NMK: Republic of North Macedonia; MED: Mediterranean; non-MED: not Mediterranean; IQR= interquartile range; SD= standard deviation. [2] The Kruskal-Wallis test was used to assess differences between countries. [3] The Mann-Whitney U test was used to assess differences between the MED group and non-MED group of countries.
